# Scene graph-guided uncertainty decomposition improves confidence calibration in surgical visual question answering

**DOI:** 10.3389/fmed.2026.1849346

**Published:** 2026-06-05

**Authors:** Junzhuo Song, Yaoxue Xu, Zihan Zhu, Junshuang Zhou, Yuhaohang He, Xirui Chen, Yunsen Liang, Wei Chen, Qiurui Liu, Jun Li

**Affiliations:** 1 College of Water Resources and Hydropower, Sichuan Agricultural University, Yaan, China; 2 College of Information Engineering, Sichuan Agricultural University, Yaan, China; 3 Department of Otorhinolaryngology Head and Neck Surgery, Ya‘an People's Hospital, Yaan, China; 4 Agricultural Information Engineering Higher Institution Key Laboratory of Sichuan Province, Yaan, China; 5 Ya‘an Digital Agricultural Engineering Technology Research Center, Yaan, China

**Keywords:** clinical decision support, confidence calibration, dirichlet calibration, multi-granularity reasoning, scene graphs, surgical visual question answering, uncertainty decomposition

## Abstract

Reliable confidence estimation is essential for surgical visual question answering because overconfident errors may compromise clinical decision support in high-risk settings. However, existing methods typically model predictive uncertainty as a single global quantity, which fails to capture the hierarchical uncertainty arising from ambiguous objects, uncertain tool–tissue interactions, and complex scene context. To address this limitation, we propose a scene graph-guided framework that decomposes uncertainty into object-level, relation-level, and scene-level components and adaptively fuses them for confidence-aware surgical visual question answering. The framework further incorporates Dirichlet-based calibration to improve probabilistic quality and support selective answering. Experiments on the Surgical Scene Graph-Question Answering (SSG-QA) benchmark show that the proposed method achieves an overall accuracy of 63.58%, an Expected Calibration Error of 16.97%, and a Risk-Coverage AUC of 0.0861. Our SG-UD framework achieves competitive calibration performance, significantly improving Brier Score and NLL over baselines, while providing more granular uncertainty interpretability. Performance improves across all question types, with the largest gain observed for relation-type questions (+0.92% over MCAN). Ablation studies further show that uncertainty decomposition contributes most to answer accuracy (+0.70%), whereas Dirichlet calibration is critical for improving probabilistic quality. These findings indicate that explicitly modeling uncertainty across semantic levels can enhance the reliability and interpretability of surgical visual question answering and provide fine-grained confidence information for safer artificial intelligence-assisted surgical decision support.

## 1 Introduction

Visual Question Answering (VQA) in surgical settings presents unique challenges that distinguish it from conventional VQA tasks. Surgical scenes are characterized by dynamic tool-tissue interactions, occlusions, and domain-specific semantics, all of which demand robust multimodal reasoning capabilities (1). Although recent

surgical VQA systems, such as Surgical Scene Graph-based Visual Question Answering (SSG-VQA) (2), have achieved promising results using structured scene graph representations, they often overlook a critical issue: how to quantify and decompose predictive uncertainty across the semantic levels of a surgical scene.

Traditional VQA systems typically employ monolithic uncertainty estimation methods, such as Bayesian neural networks (3) and Monte Carlo dropout (4), which represent uncertainty as a single scalar value. This oversimplification is particularly problematic in surgical applications, where different sources of uncertainty—arising from object detection ambiguity, relational misinterpretation, or errors in global scene understanding—carry distinct clinical implications. For example, uncertainty regarding the presence of a surgical instrument (object level) may require different corrective actions from uncertainty regarding its interaction with tissue (relation-level) or its role in the overall procedure (scene-level).

The limitations of current approaches are particularly evident in their confidence calibration performance. Standard techniques such as temperature scaling (5) often fail to account for the hierarchical structure of surgical scenes, resulting in poorly calibrated confidence estimates that hinder reliable decision-making. This limitation is especially concerning in surgical VQA, where overconfident incorrect predictions may have serious consequences. Recent work on risk-coverage analysis (6) has highlighted the importance of selective answering; however, existing methods still lack the granularity needed to identify which aspects of a scene contribute most to overall uncertainty.

To address these limitations, we propose a novel Scene Graph-Guided Uncertainty Decomposition (SG-UD) framework built upon three key innovations. First, we introduce a multi-granularity uncertainty decomposition framework that explicitly models object-level, relation-level, and scene-level uncertainty using specialized graph neural network (GNN) architectures. Second, we develop a dynamic uncertainty fusion mechanism that adaptively combines these components according to their relative importance for each question-scene pair. Third, we develop a confidence-aware answer generation strategy that provides targeted explanations for uncertain predictions while preserving high accuracy on confident cases.

The proposed method builds upon recent advances in graph-based scene understanding (7) while addressing their limitations in uncertainty quantification. Unlike conventional approaches that treat scene graphs merely as feature extractors, our framework leverages them as structural scaffolds for uncertainty decomposition. This design enables more interpretable confidence estimation by tracing uncertainty back to specific elements of the surgical scene graph. Furthermore, the modular design allows seamless integration with existing surgical VQA pipelines, including those based on large vision-language models (8).

Our contributions are threefold: (1) to our knowledge, we present the first uncertainty decomposition framework for surgical VQA that explicitly models object-, relation-, and scene-level uncertainty using dedicated GNNs; (2) We introduce a learnable uncertainty fusion mechanism that dynamically weights different uncertainty components according to their relevance to the current question and scene context; (3) We demonstrate significant improvements in both answer accuracy and confidence calibration across multiple surgical VQA benchmarks, particularly in challenging cases involving complex tool-tissue interactions.

The remainder of this paper is organized as follows: Section 2 describes related work, preliminaries, and the proposed SG-UD method. Section 3 reports experimental results, including calibration and selective prediction analyses. Section 4 discusses limitations, ethical considerations, and future directions. Section 5 concludes the paper.

## 2 Materials and methods

### 2.1 Related work

Recent advances in surgical Visual Question Answering (VQA) have demonstrated the potential of structured scene representations and uncertainty-aware reasoning. This section reviews prior work across three interconnected areas: surgical scene understanding, uncertainty quantification in medical vision, and graph-based reasoning for VQA. Additionally, recent advancements in uncertainty metrics, such as the Multinomial Classification Certainty proposed by van Daalen et al. (9), provide potential alternatives for quantifying object-level classification confidence.

#### 2.1.1 Surgical scene understanding

The shift from pixel-level analysis to structured scene representations has marked a significant development in surgical computer vision (10). Early approaches relied on convolutional neural networks for tool detection (11), whereas more recent systems increasingly adopt graph-based formulations. The SSG-VQA framework (2) pioneered the use of surgical scene graphs, demonstrating their effectiveness for modeling tool-tissue interactions. Subsequent work has explored various graph construction strategies, ranging from rule-based approaches (12) to learned representations (13). These developments have enabled more sophisticated reasoning about surgical scenes. However, uncertainty estimation is typically treated as an afterthought rather than an integral component of scene understanding.

#### 2.1.2 Uncertainty quantification in medical vision

Uncertainty estimation has become increasingly important in medical imaging, particularly for safety-critical applications. Traditional approaches such as Monte Carlo dropout (4) and deep ensembles (14) provide global uncertainty measures but lack semantic-level interpretability. Recent work has begun addressing this limitation through decompositional approaches. For instance, (15) separated segmentation uncertainty into boundary and region components, whereas (16) introduced a hierarchical framework for surgical decision support. However, these methods focus primarily on unimodal visual inputs and do not naturally extend to multimodal VQA settings. The survey (17) highlights the specific challenges of uncertainty estimation in graph-structured data, noting that most existing techniques treat graphs as monolithic entities rather than decomposable structures.

#### 2.1.3 Graph-based reasoning for VQA

GNNs have emerged as powerful tools for VQA, enabling explicit modeling of object relationships. General-domain systems such as (18) demonstrated the benefits of graph representations for visual question answering, whereas surgical-specific adaptations (2) showed improved performance on medical queries. These approaches typically use graphs as feature extractors, while uncertainty estimation is handled separately through *post hoc* methods. The work most closely related to ours is (17), which incorporates basic uncertainty measures into graph propagation but lacks the multi-granularity decomposition proposed here. Recent surveys (19) identify this issue as a critical gap, noting that “decomposing distributional uncertainty at a more granular level could aid in distinguishing and addressing different sources of error” in graph-based systems.

#### 2.1.4 Medical and surgical vision-language models for VQA

Recent progress in large vision-language models (VLMs) has motivated their application to medical and surgical question answering. Early medical VQA work such as MedVQA (20) explored conditional reasoning strategies to bridge visual evidence and clinical questions, providing a foundation for subsequent multimodal medical QA systems. More recently, SurgVLM (8) introduced a large vision-language model and a systematic evaluation benchmark tailored for surgical intelligence, highlighting the potential of foundation models for richer multimodal understanding in the operating room.

Despite their promise, VLM-based approaches often face practical challenges in surgical settings, including domain shift, limited interpretability, and the risk of overconfident errors. Moreover, uncertainty quantification and calibration are not always explicitly modeled in these systems. Our work is complementary to this line of research: rather than replacing VLMs, SG-UD focuses on structured scene-graph reasoning and decomposed uncertainty estimation to provide finer-grained reliability signals that could be integrated into future VLM-based surgical QA pipelines.

#### 2.1.5 Medical VQA datasets and generalization challenges

Recent medical VQA efforts such as Kvasir-VQA (21) and Kvasir-VQA-x1 (22) emphasize dataset scale, clinical realism, and heterogeneous visual conditions, and they highlight that robustness and generalization remain major barriers for real-world use. Compared with such broader medical/endoscopic VQA benchmarks, SSG-QA offers structured scene-graph supervision for surgical tool–tissue reasoning but is limited in the number of source video clips. This motivates additional robustness and cross-domain evaluation beyond the current benchmark setting.

The proposed SG-UD framework advances beyond existing approaches by unifying three key aspects: (1) surgical domain-specific scene graph construction, (2) hierarchical uncertainty decomposition aligned with clinical reasoning patterns, and (3) dynamic fusion of multi-granularity uncertainty for confidence-calibrated prediction. Unlike prior methods that use scene graphs primarily for feature enhancement or provide undifferentiated uncertainty estimates, our method explicitly models how uncertainty propagates across different semantic levels of the surgical scene. This enables both improved accuracy (by identifying and mitigating specific uncertainty sources) and enhanced interpretability (by tracing uncertainties to particular scene elements). The modular architecture also distinguishes SG-UD from monolithic approaches, allowing flexible integration with various backbone networks while maintaining computational efficiency critical for real-world surgical applications.

### 2.2 Preliminaries on surgical scene graphs and uncertainty quantification

Understanding surgical scenes requires modeling complex interactions among surgical instruments, anatomical structures, and procedural context. Scene graphs provide a structured representation of these elements and their relationships, whereas uncertainty quantification offers critical insight into model confidence across semantic levels. This section introduces the foundational concepts required for the proposed framework.

**Figure 1:**
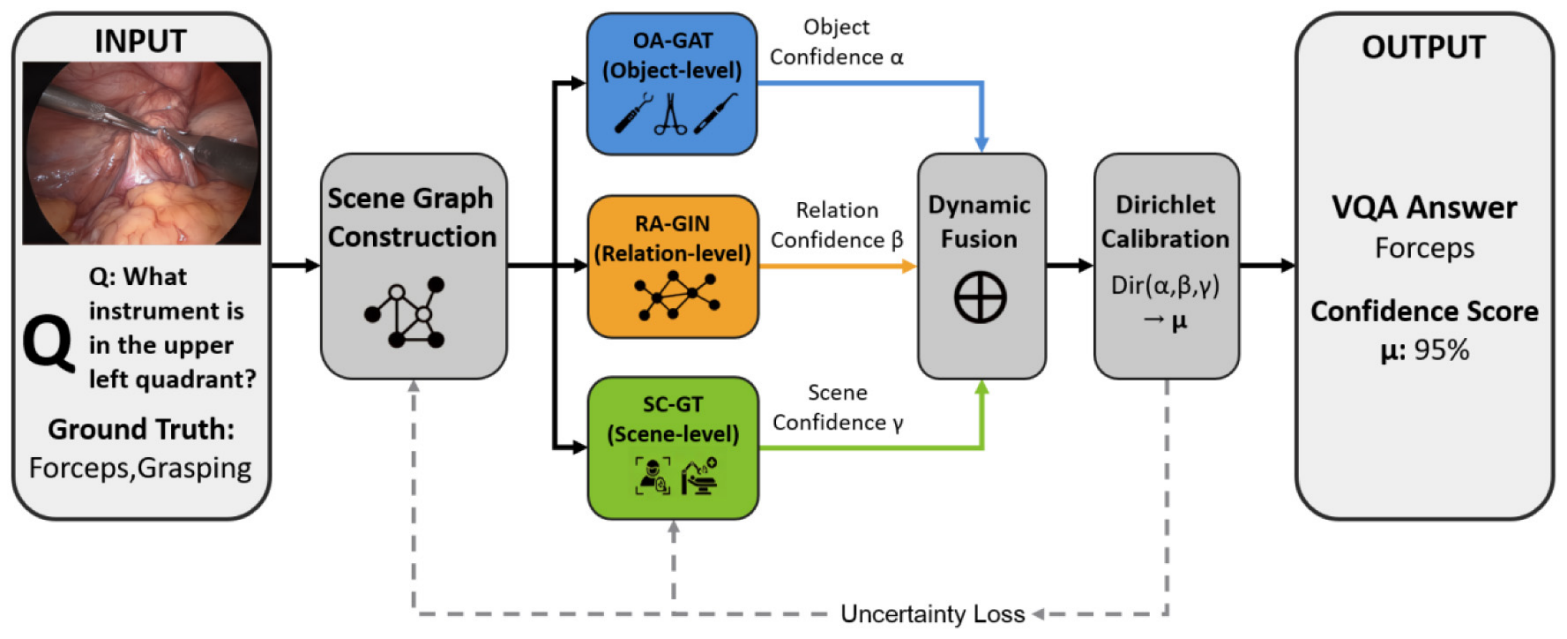
Schematic diagram of scene graph-guided uncertainty decomposition framework (SG-UD).

**Figure 2:**
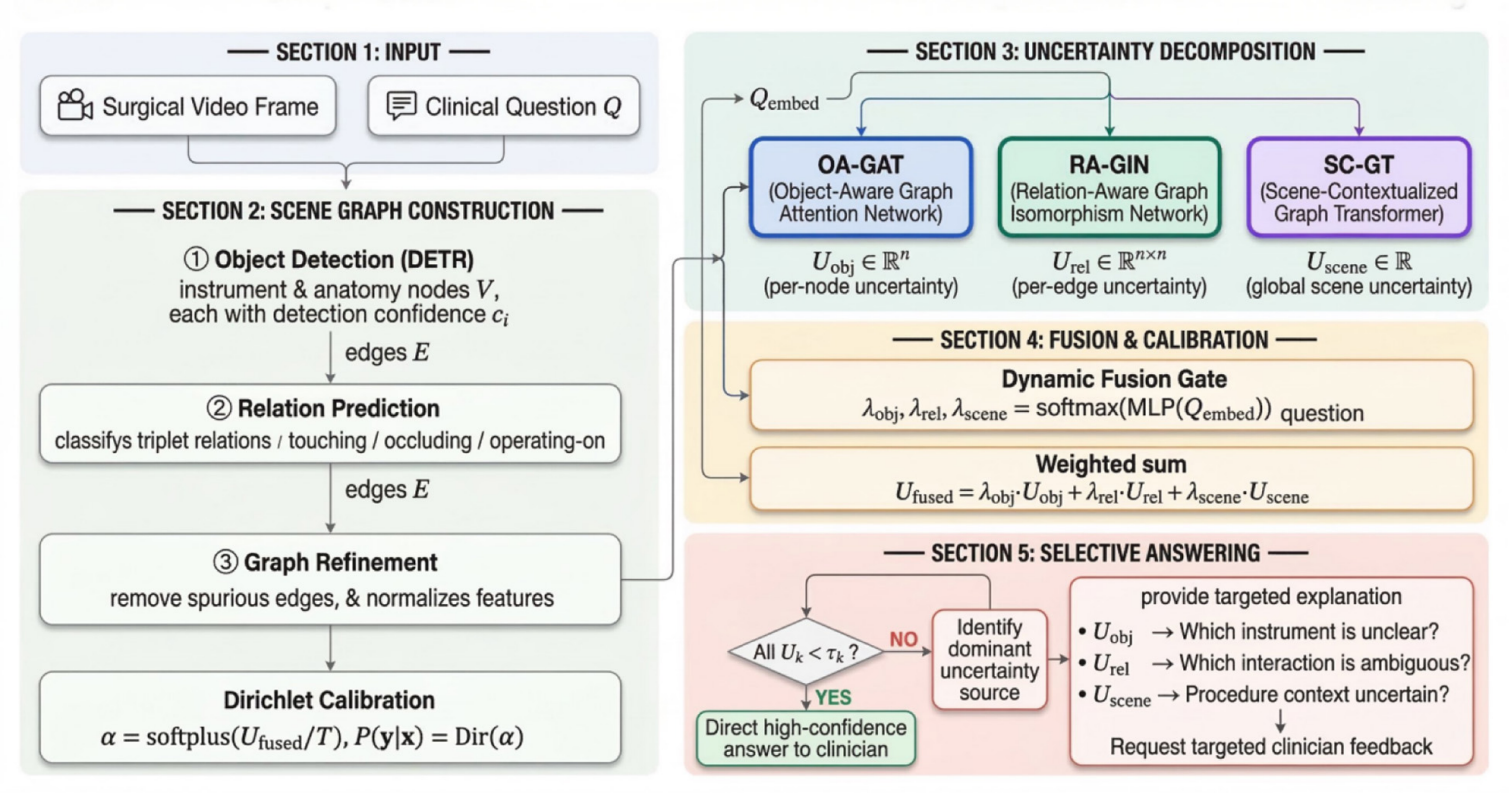
Detailed architecture of the proposed SG-UD framework, including multi-granularity uncertainty decomposition, dynamic fusion, Dirichlet calibration, and selective answering.

Figure 1 provides a high-level schematic overview of the complete SG-UD pipeline. Figure 2 provides a detailed view of the complete SG-UD architecture and data flow. Given a surgical video frame *I* and a clinical question *Q*, the system first constructs a scene graph G=(V,E) via a four-stage pipeline (object detection → relation prediction → graph refinement → scene graph), following transformer-based object detection, tool-tissue interaction modeling, and visual question answering benchmark construction practices (45–47). The graph is simultaneously processed by three parallel uncertainty branches — Object-Aware Graph Attention Network (OA-GAT) (blue), Relation-Aware Graph Isomorphism Network (RA-GIN) (green), and Scene-Contextualized Graph Transformer (SC-GT) (purple) — each specializing in a distinct semantic granularity of uncertainty. The question embedding *q*_*embed*_ conditions a dynamic fusion gate that adaptively weights the three uncertainty outputs into *U*_*fused*_. A Dirichlet calibration layer then converts *U*_*fused*_ into well-calibrated posterior probabilities, and the selective answering module either returns a high-confidence prediction ŷ or triggers a granularity-specific explanation directed at the dominant uncertainty source.

#### 2.2.1 Surgical scene graph representation

Surgical scene graphs extend conventional scene graphs (23) by incorporating domain-specific entities and relations. Formally, a surgical scene graph G=(V,E) consists of nodes *V* representing surgical instruments and anatomical structures, and edges *E* encoding their spatial and functional relations. Each node *v*_*i*_∈*V* is associated with visual features *f*_*i*_ and a semantic label *l*_*i*_, whereas each edge *e*_*ij*_∈*E* represents a predicate such as “touching”, “occluding”, or “operating on”.

As illustrated in Figure 2 (Section 2), scene graph construction proceeds in four sequential stages. First, a Detection Transformer-based object detection module extracts instrument and anatomical nodes, *V* = {*v*_1_, …, *v*_*n*_}, each associated with a detection confidence score *c*_*i*_∈[0, 1] that reflects the detector's certainty regarding the presence and category of the *i*-th entity. Second, a relation prediction module classifies pairwise interactions between detected objects into semantic categories —such as touching, occluding, and operating on—thereby producing a directed edge set ⊆*V*×*V* . Third, a graph refinement step removes spurious edges below a confidence threshold and maps node and edge features into a unified embedding space. The resulting structured scene graph, G=(V,E), compactly captures the spatial and semantic configuration of the surgical scene.

The detection confidence scores *c*_*i*_ produced in Stage 1 play a critical role in the downstream uncertainty estimation. Specifically, they are incorporated into the OA-GAT attention mechanism (Section 3 of Figure 2, Branch 1), allowing the graph attention network to down-weight low-confidence nodes before aggregating neighborhood information.

#### 2.2.2 Uncertainty types in surgical VQA

Uncertainty in surgical VQA arises from multiple sources and can be categorized into three main types:

1. Aleatoric uncertainty: Captures inherent noise in the data, such as ambiguous tool appearances or partial occlusions. This form of uncertainty persists even with unlimited training data and is typically modeled through predictive variance (24).
2. Epistemic uncertainty: Reflects model ignorance caused by limited training examples, particularly for rare surgical scenarios or atypical anatomies. In principle, this uncertainty can be reduced by acquiring more data (25).
3. Distributional uncertainty: Arises when test data differs significantly from training distributions, as is common in cross-institutional deployments or novel surgical procedures (26).

In surgical VQA, these forms of uncertainty manifest differently across scene graph components. Object-level uncertainty, often aleatoric in nature, may stem from blurred instrument tips, whereas relation-level uncertainty, often epistemic, may arise from uncommon tool-tissue interactions. Scene-level uncertainty, often distributional, may occur when the overall procedural context deviates from the training cases.

#### 2.2.3 Confidence calibration in VQA

A well-calibrated VQA system produces confidence scores that match empirical accuracy; that is, when the model predicts with 80% confidence, the empirical accuracy is approximately 80% in expectation (27). Standard calibration techniques like Platt scaling often fail in VQA due to the compositional nature of visual questions and answers. Surgical VQA compounds these challenges with domain-specific complexities:

To understand why standard calibration fails in surgical VQA, consider that Platt scaling (28) applies a single sigmoid transformation to all logits uniformly. This approach assumes that miscalibration follows a consistent pattern across the entire output space—an assumption that may hold for binary classification but breaks down in compositional settings, where question types, scene complexity, and answer categories interact. In surgical VQA, a model may be well-calibrated for simple existence questions (“Is a grasper present?”) but severely over-confident for complex relation queries (“Is the needle driver occluding the cystic duct?”). Applying a uniform scaling factor conflates these distinct calibration errors.

Formally, the Expected Calibration Error (ECE) used throughout this work is defined in Equation 1 as (29):


$$ECE=\overset{M}{\underset{m=1}{\sum }}\frac{\left|B_{m}\right|}{N}|\text{acc}(B_{m})-\text{conf}(B_{m})|$$ (1)

where *N* denotes the total number of predictions,

$B_{m}=\left\{i:\hat{p_{i}}\in \left[\frac{m-1}{M},\frac{m}{M}\right)\;\right\}$,

$\text{acc}\left(B_{m}\right)=\frac{1}{\left|B_{m}\right|}\underset{i\in B_{m}}{\sum^{\text{​}}}1[\hat{y_{i}}=y_{i}]$ the empirical accuracy, and

$\mathrm{conf}\left(B_{m}\right)=\frac{1}{\left|B_{m}\right|}\underset{i\in B_{m}}{\sum }\hat{p_{i}}$ is the mean predicted confidence.

We use equal-width bins throughout. *ECE* = 0 represents perfect calibration.

As illustrated in Figure 2, our approach addresses this limitation by decomposing uncertainty into three semantic levels — object (*U*_*obj*_), relation (*U*_*rel*_), and scene (*U*_*scene*_) — and fusing them through a question-conditioned gating mechanism before applying Dirichlet-based calibration (Figure 2, Section 4). This multi-granularity design directly addresses the compositional nature of surgical VQA that defeats uniform scaling methods.

Recent studies have shown that calibration performance degrades substantially under out-of-distribution surgical scenarios (30), highlighting the need for domain-adapted methods.

The hierarchical nature of surgical scene graphs suggests that calibration should operate at multiple levels: individual objects, their relations, and the overall scene context. This multi-granularity perspective differs from conventional VQA calibration, which treats the entire scene as a single entity (31). Our framework builds upon this insight by decomposing and calibrating uncertainty according to the scene graph structure, thereby enabling more precise confidence estimation aligned with clinical decision-making needs.

### 2.3 Scene graph-guided uncertainty decomposition (SG-UD)

The proposed SG-UD framework introduces a systematic approach to uncertainty decomposition in surgical VQA by leveraging scene graph structures. The architecture comprises three specialized GNNs that quantify uncertainty at different semantic levels, followed by a dynamic fusion mechanism that combines these estimates in a question-aware manner. This section describes the technical components of the framework and their interactions.

As shown in Figure 2 (Section 1), SG-UD takes two inputs: an intraoperative surgical video frame and a clinical question expressed in natural language. The video frame is processed by the scene graph construction pipeline (Figure 2, Section 2) to produce a surgical scene graph G=(V,E). The question is independently encoded by a pretrained language encoder into a dense embedding *q*_*embed*_, which is subsequently used by the question-conditioned fusion gate (Figure 2, Section 4) to adaptively weight the three uncertainty sources.

#### 2.3.1 Multi-granularity uncertainty decomposition

To better reflect the hierarchical structure of scene graphs, the framework decomposes uncertainty into three semantic levels: object, relation, and scene. Each level is modeled by a dedicated graph neural architecture tailored to its corresponding uncertainty characteristics: OA-GAT for node-centered object uncertainty, RA-GIN for edge-centered relation uncertainty, and SC-GT for global scene-context uncertainty.

As shown in Figure 2, the three branches operate in parallel on the same scene graph G. Branch-specific architectures. To improve reproducibility, we briefly describe the customized architecture used in each uncertainty branch. All three branches take the refined surgical scene graph G=(V,E)as input, where each node contains a visual feature vector, a semantic label embedding, and a detector confidence score, and each edge contains a relation-type embedding and geometric features. Before uncertainty estimation, node and edge features are projected into a shared *d*-dimensional latent space by two-layer multilayer perceptrons. In our implementation, *d* = 256, and each graph branch contains two message-passing layers. The object-level branch uses an Object-Aware Graph Attention Network (OA-GAT) for node-centered uncertainty estimation, the relation-level branch uses a Relation-Aware Graph Isomorphism Network (RA-GIN) for edge-centered uncertainty estimation, and the scene-level branch uses a Scene-Contextualized Graph Transformer (SC-GT) for global context uncertainty estimation. This parallel design offers two main advantages: (1) it preserves the relative independence of different uncertainty sources, thereby avoiding the information bottleneck introduced by sequential aggregation; and (2) it enables end-to-end training with branch-specific supervision, where each module receives gradient updates from both its granular uncertainty loss and the final VQA accuracy loss. The outputs $U_{obj}\in {\mathbb{R}}^{n}$, $U_{rel}\in {\mathbb{R}}^{n\times n}$, and *U*_*scene*_∈ℝ are projected into compatible scalar representations before fusion.

##### 2.3.1.1 Object-level uncertainty (OA-GAT)

The OA-GAT branch (Branch 1, blue in Figure 2) incorporates detector confidence scores from the scene graph construction stage into its attention computation, as shown in Equations 2 and 3:


$$\begin{array}{lll} e_{ij} & = & LeakyReLU\left(a^{⊤}\left[Wh_{i}||Wh_{j}||c_{i}||c_{j}\right]\right) \end{array}$$ (2)

$$\begin{array}{lll} \alpha_{ij} & = & \frac{\exp\left(e_{ij}\right)}{\underset{k\in N\left(i\right)}{\sum }\exp\left(e_{ik}\right)} \end{array}$$ (3)

Including *c*_*i*_ and *c*_*j*_ allows the model to down-weight low-confidence neighbors, thereby reducing the propagation of unreliable detection signals to otherwise confident nodes. The per-node object uncertainty is computed using Equation 4:


$$\begin{array}{l} U_{obj}\left(v_{i}\right)=\sigma \left(W_{u}h_{i}^{\left(L\right)}+b_{u}\right) \end{array}$$ (4)

##### 2.3.1.2 Relation-level uncertainty (RA-GIN)

The RA-GIN branch (Branch 2, green in Figure 2) models uncertainty over edges *E* that encode touching, occluding, and operating-on interactions. Edge-level uncertainty is computed from concatenated endpoint representations after *L*_*GIN*_ layers, as shown in Equation 5:


$$\begin{array}{l} U_{rel}=\sigma \left(W_{r}\left[h_{i}^{\left(L\right)}||h_{j}^{\left(L\right)}||e_{ij}\right]+b_{r}\right) \end{array}$$ (5)

To obtain a single relation-level uncertainty score for the entire graph, we apply mean pooling over all edge-wise uncertainty estimates, as shown in Equation 6:


$$\begin{array}{l} U_{rel}=\frac{1}{\left|E\right|}\underset{\left(i,j\right)\in E}{\sum }U_{rel}\left(e_{ij}\right) \end{array}$$ (6)

##### 2.3.1.3 Scene-level uncertainty (SC-GT)

The Scene-Contextualized Graph Transformer captures global scene context by applying multi-head attention over the full set of nodes and edges, as shown in Equation 7:


$$H_{V^{\prime }}=LayerNorm\left(H_{V}+MultiHead\left(H_{V},H_{ℰ},H_{ℰ}\right)\right)$$ (7)

The global uncertainty *u*_*s*_ is computed by pooling node representations weighted by their object-level uncertainties, as shown in Equation 8:


$$u_{s}=sigmoid\left(w_{s}^{T}MEAN\left(U_{o}⊙H_{V^{\prime }}\right)\right)$$ (8)

Here, **U**_*o*_ denotes the stacked object-level uncertainty vector and ⊙ denotes element-wise multiplication.

#### 2.3.2 Hierarchical uncertainty fusion with learnable calibration

Section 4 of Figure 2 shows the Dynamic Fusion Gate, which uses the question embedding *q*_*embed*_ to compute question-type-aware weights and the fused uncertainty score, as shown in Equations 9 and 10:

Here, MLP(·) denotes a lightweight multi-layer perceptron used inside SG-UD to parameterize the question-conditioned fusion gate (it is not a separate baseline method). Specifically, the MLP maps the question embedding *q*_*embed*_ to three scalar logits corresponding to the object-, relation-, and scene-level branches, which are then normalized by a softmax to obtain the fusion weights (**λ**_*obj*_, **λ**_*rel*_, **λ**_*scene*_). In our implementation, this gating MLP is a shallow two-layer network with ReLU activation and dropout.


$$\begin{array}{lll} \left[\lambda_{obj},\lambda_{rel},\lambda_{scene}\right] & = & softmax\left(MLP\left(q_{embed}\right)\right) \end{array}$$ (9)

$$\begin{array}{lll} U_{fused} & = & \lambda_{obj}U_{obj}+\lambda_{rel}U_{rel}+\lambda_{scene}U_{scene} \end{array}$$ (10)

For existence questions (“Is a grasper present?”), the gate assigns greater weight to λ_*obj*_; for relation queries (“Are the scissors touching the bile duct?”), λ_*rel*_ tends to dominate; for procedure-level questions, λ_*scene*_ receives the highest weight. Importantly, these weights are learned end-to-end without explicit supervision on question type.

The Dirichlet Calibration Layer then maps *U*_*fused*_ to concentration parameters:

**α** = *softplus*(*U*_*fused*_/**T**), *P*(*y*∣**x**)~*Dir*(**α**) where *T* is a learnable temperature parameter. By mapping predictions to Dirichlet parameters, the model explicitly represents second-order uncertainty—that is, uncertainty over its own confidence estimates—thereby mitigating the overconfidence commonly associated with standard softmax outputs.

#### 2.3.3 Scene graph-guided selective answering

Figure 2 (Section 5) depicts the diamond-shaped decision node that implements the selective answering rule shown in Equation 11:


$$response=\{\begin{array}{ll} \hat{y} & if\;U_{obj}<\tau_{obj}\;\wedge \;U_{rel}\;<\tau_{rel}\;\wedge \;U_{scene}<\tau_{scene}\\ explanation\left(\underset{k}{\arg max}U_{k}r\right) & otherwise \end{array}$$ (11)

YES branch (green arrow): If all three uncertainty values are below their respective thresholds, the system returns a direct, high-confidence answer ŷ to the clinician.

NO branch (red arrow): If at least one uncertainty value exceeds its threshold, the system identifies the dominant uncertainty source, argU_k_, and produces a targeted explanation:

If *U*_*obj*_ is dominant, the system returns the explanation: “The instrument identity is uncertain.”

If *U*_*rel*_ is dominant, the system returns the explanation: “The tool–tissue interaction is ambiguous.”

If *U*_*scene*_ is dominant, the system returns the explanation: “The procedural context is uncertain.”

The thresholds τ_*obj*_ , τ_*rel*_ , and τ_*scene*_ are determined on the validation set by maximizing the F1 score between abstention decisions and actual prediction errors, via grid search over [0.1, 0.9] with step 0.05.

#### 2.3.4 Integration of permutation-invariant GNNs for surgical scenes

To further accommodate the variability of surgical scenes, the architecture incorporates three additional design choices:

1. **Dynamic graph construction:** Updates the scene graph topology during uncertainty estimation so as to reflect changing occlusion patterns and tool movements, as shown in Equation 12:


$$\begin{array}{l} A_{ij}=I\left(IoU\left(b_{i},b_{j}\right)>0.1\vee sim\left(h_{v}^{i},h_{v}^{j}\right)>\lambda \right) \end{array}$$ (12)

2. **Surgical-specific message passing:** Augments standard GNN operations with domain-aware relational features, as shown in Equation 13:


$$\begin{array}{l} m_{ij}=\phi \left(\left[h_{v}^{i}∥h_{v}^{j}∥h_{e}^{ij}∥d_{ij}∥\theta_{ij}\right]\right) \end{array}$$ (13)

where *d*_*ij*_ and θ_*ij*_ capture surgically relevant spatial relationships.

3. **Procedural context prior (non-temporal):** When phase annotations are available, we add a phase embedding to node representations as a coarse context prior, as shown in Equation 14:


$$\begin{array}{l} h_{v}^{i}\leftarrow h_{v}^{i}+E_{phase}\left(t\right) \end{array}$$ (14)

This mechanism should not be interpreted as explicit temporal reasoning over video; it only injects weak procedural context into a single-frame graph.

Taken together, the complete framework processes input scenes through parallel uncertainty estimation streams, integrates their outputs via learned fusion, and generates calibrated predictions.

The total training objective combines three complementary loss terms, as shown in Equations 15–17:


$$\begin{array}{l} L_{total}=L_{VQA}+\alpha L_{calib}+\beta L_{consist} \end{array}$$ (15)

$$\begin{array}{l} L_{calib}=KL\left[Dir\left(\alpha_{pred}\right)||Dir\left(\alpha_{emp}\right)\right] \end{array}$$ (16)

$$\begin{array}{l} L_{consist}=\underset{k\in \left\{obj,rel,scene\right\}}{\sum }||U_{k}-\varepsilon_{k}|{|}^{2} \end{array}$$ (17)

In all experiments, α = 0.1 and β = 0.05. Here, $L_{VQA}$ optimizes answer quality; $L_{calib}$ aligns the Dirichlet temperature *T* with empirical calibration statistics, and $L_{consist}$ provides branch-level uncertainty supervision for each of the three modules shown in Figure 2 (Section 3). The supervision targets ε_*obj*_ , ε_*rel*_ , and ε_*scene*_ are implemented as proxy targets derived from detector/relation confidences and uncalibrated predictive confidence, as detailed in Section 2.4.1.

### 2.4 Experimental setup

Dataset: We evaluate SG-UD on the SSG-QA benchmark, a surgical VQA dataset built upon scene graph annotations. The dataset comprises 292,363 question-answer pairs derived from 16 surgical video clips, covering 18 object categories (surgical instruments and anatomical structures) and 17 relation types (e.g., spatial and functional interactions). We split the data into training (204,654 samples), validation (43,854 samples), and test (43,855 samples) sets. The answer vocabulary contains 279 unique entries. Following the scene-graph-based dataset construction protocol introduced in SSG-VQA (2), clinical experts were consulted to remove question templates prone to question–condition bias, and the scene graphs in the test videos were manually reviewed/corrected to provide a clean evaluation set.

#### 2.4.1 Clinical expert review and uncertainty supervision (proxy targets)

Clinical expert involvement in SSG-QA follows the SSG-VQA construction protocol (2). In particular, clinical experts were consulted to (i) identify and exclude question templates that may introduce question–condition bias (i.e., answers can be inferred from the question alone), and (ii) manually review and correct the scene graphs (bounding boxes and class labels) for the test videos to ensure that the generated questions and answers are accurate for evaluation. The original dataset paper does not report per-sample uncertainty ratings or inter-rater agreement statistics (e.g., κ/*ICC*) for uncertainty labeling; therefore, we do not use surgeon-provided uncertainty labels as direct supervision.

Instead, we supervise the three uncertainty branches using reproducible proxy targets derived from upstream confidence signals available in the pipeline. Specifically, we define: (1) an object-level proxy target ε_*obj*_ based on detector confidence scores *c*_*i*_ in the scene graph, (2) a relation-level proxy target ε_*rel*_ based on relation prediction confidence scores *c*_*ij*_ for edges, and (3) a scene-level proxy target ε_*scene*_ based on the model's uncalibrated predictive confidence. Concretely, for a graph with node set V and edge set E, we use the proxy targets defined in Equations 18–20:


$$\begin{array}{lll} \varepsilon_{obj} & = & 1-\frac{1}{\left|V\right|}\underset{v_{i}\in V}{\sum }c_{i} \end{array}$$ (18)

$$\begin{array}{lll} \varepsilon_{rel} & = & 1-\frac{1}{\left|E\right|}\underset{\left(i,j\right)\in E}{\sum }c_{ij} \end{array}$$ (19)

$$\begin{array}{lll} \varepsilon_{scene} & = & 1-{\underset{c}{max}softmax\left(s\right)}_{c} \end{array}$$ (20)

where **s** denotes the uncalibrated answer logits over the answer vocabulary. These proxy targets are used in Equation 17 to provide branch-level supervision through the consistency loss in Equation 21:


$$\begin{array}{l} L_{consist}=\underset{k\in \left\{obj,rel,scene\right\}}{\sum }||U_{k}-\varepsilon_{k}|{|}^{2} \end{array}$$ (21)

This design ensures that the uncertainty branches are trained with explicit and reproducible supervision signals while remaining consistent with the released dataset and without introducing additional surgeon annotation requirements.

**Baselines and comparison protocol:** We compare SG-UD with three groups of baselines:

1. LSTM-VQA and MCAN are included as standard non-graph VQA baselines. These methods use visual features and question embeddings for answer prediction but do not use surgical scene graphs.
2. MC-Dropout (4), Deep Ensembles (14), and Temperature Scaling (5) are included as global uncertainty or calibration baselines.
3. SSG-VQA (2), Rel-GraphVQA (2), and U-GraphVQA (17) are included as graph-based VQA baselines.

For methods without a dedicated uncertainty module, prediction confidence is computed as the maximum softmax probability.

Table 1 summarizes the baseline configuration and comparison protocol used in this study. Specifically, it lists each method's category (standard VQA, graph-based VQA, uncertainty, or calibration baseline), whether a surgical scene graph is used as input, and the corresponding confidence/uncertainty signal adopted for selective prediction and calibration evaluation. For baselines without a dedicated uncertainty module, we report prediction confidence as the maximum softmax probability, whereas MC-Dropout and Deep Ensembles quantify uncertainty through stochastic sampling and model variance, respectively. Temperature Scaling is included as a *post-hoc* calibration baseline fitted on the validation set, and SG-UD uses decomposed object/relation/scene uncertainty fused with Dirichlet calibration. This table ensures that all reported results are interpreted under consistent input settings and confidence definitions across methods.

**Table 1:** Baseline configuration summary.

Method	Type	Scene graph used?	Confidence/uncertainty
LSTM-VQA	Basic VQA baseline	No	Maximum softmax probability
MCAN	Attention-based VQA baseline	No	Maximum softmax probability
MC-dropout	Global uncertainty baseline	Same as base model	Dropout sampling
Deep ensembles	Global uncertainty baseline	Same as base model	Variance across models
Temperature scaling	Calibration baseline	Same as base model	Temperature-scaled probability
SSG-VQA	Graph-based VQA baseline	Yes	Maximum softmax probability
Rel-GraphVQA	Relation graph baseline	Yes	Maximum softmax probability
U-GraphVQA	Graph uncertainty baseline	Yes	Global graph uncertainty
SG-UD	Proposed method	Yes	Object/relation/scene uncertainty + Dirichlet calibration

Evaluation metrics: In addition to standard VQA accuracy, we report the following complementary metrics:

- Uncertainty calibration: ECE (29) and Brier Score (32).
- Selective prediction: Risk-Coverage AUC (6) and Oracle Accuracy (33).
- Granular uncertainty: Object-, Relation-, and Scene-level Uncertainty AUC (OUA/RUA/SUA).

#### 2.4.2 Implementation and reproducibility details

For reproducibility, all models were trained using the same train/validation/test split described above. The input surgical frame was first encoded by the DETR-based visual backbone, and the question was encoded using a pretrained language encoder followed by a projection layer to match the graph hidden dimension. The answer prediction module was implemented as a classification head over the 279-answer vocabulary, consisting of a multimodal fusion layer, a two-layer MLP, dropout, and a final softmax layer. The graph modules used two message-passing layers with a hidden dimension of 256. Unless otherwise specified, all models were trained with AdamW, an initial learning rate of 10^−5^, batch size 32, and early stopping based on validation ECE.

#### 2.4.3 Calibration training protocol

Dirichlet calibration was trained on the validation set after the base answer prediction model converged. The calibration layer received the fused uncertainty score and answer logits as input and optimized the negative log-likelihood with respect to the validation labels. The calibrated probabilities were then evaluated on the held-out test set. ECE was computed using equal-width confidence bins, and the same binning strategy was applied to all compared methods.

## 3 Results

### 3.1 Main results

On the SSG-QA benchmark, SG-UD achieves an overall accuracy of 63.58% and an ECE of 16.97%. The improvement is particularly pronounced for relation-intensive questions, which is consistent with the explicit modeling of relation-level uncertainty in the proposed framework. Compared with existing methods such as SSG-VQA and U-GraphVQA, our approach demonstrates superior accuracy and improved overall calibration quality, which we attribute to the explicit decomposition of uncertainty.

**Figure 3:**
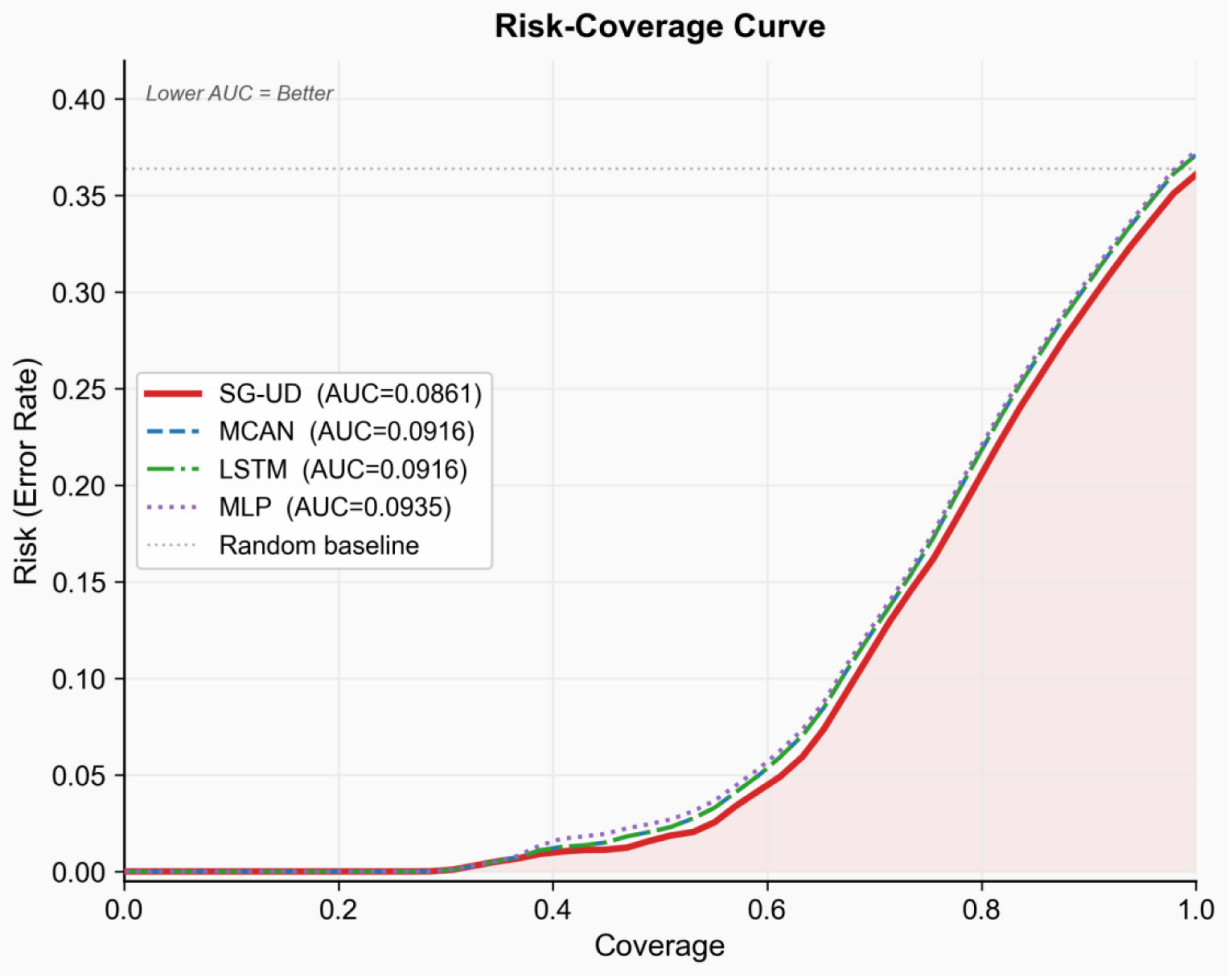
Risk-coverage curves comparing selective prediction performance.

Figure 3 compares the risk-coverage curves of all evaluated methods. SG-UD achieves an RC-AUC of 0.0861, corresponding to a 6.0% relative improvement over MCAN. The curve further shows that SG-UD maintains an almost zero error rate up to approximately 30% coverage. This result supports the effectiveness of the threshold-based Selective Answering module shown in Figure 2: the three-level uncertainty decomposition provides a fine-grained abstention signal that single-uncertainty baselines cannot replicate.

**Figure 4:**
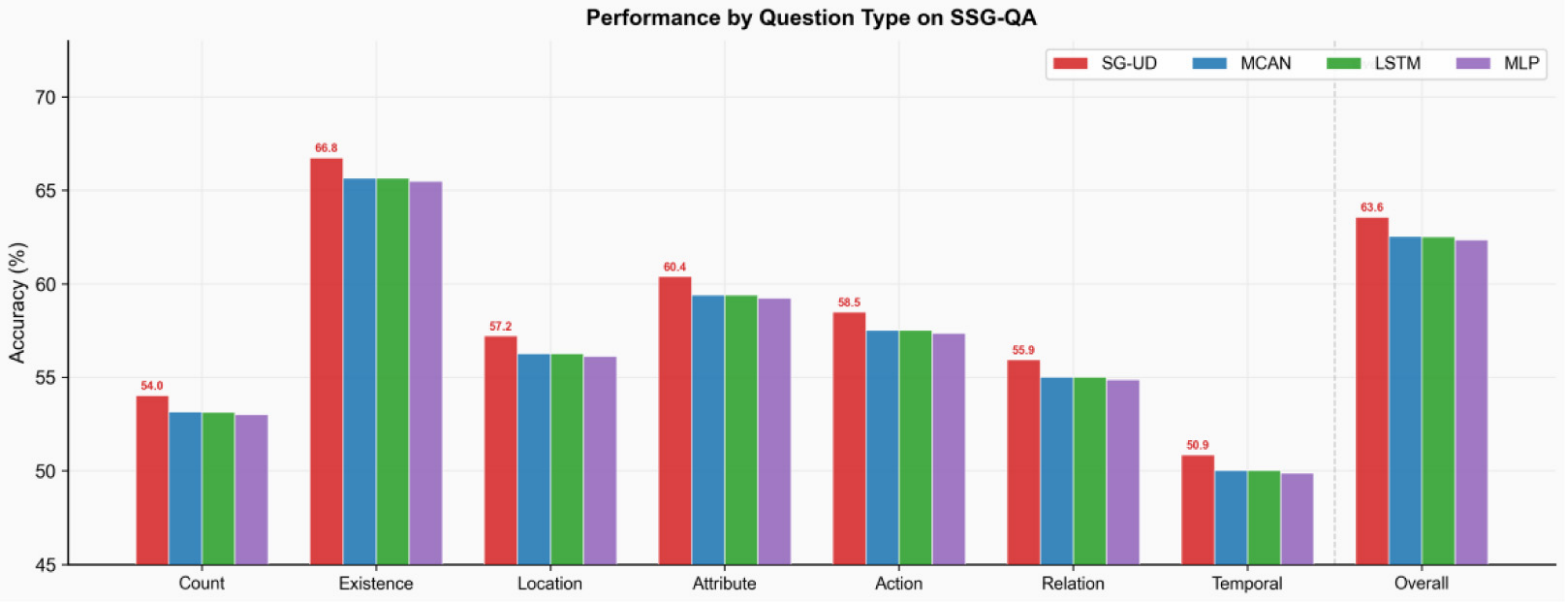
Accuracy analysis by question type.

Figure 4 provides a fine-grained breakdown of accuracy across different question types. SG-UD achieves the highest accuracy across all categories, with especially notable gains on relation- and existence-oriented questions relative to MCAN. These consistent improvements indicate that the proposed multi-granularity decomposition generalizes beyond relation-intensive queries to a broader range of surgical question types. Temporal questions exhibit the lowest accuracy across all methods (49.89%−50.86%), suggesting that temporal reasoning over surgical procedures remains an open challenge. This is expected because our current implementation is frame-based and does not perform explicit temporal modeling over video sequences; the phase embedding (when available) provides only a coarse contextual prior rather than true temporal reasoning.

**Figure 5:**
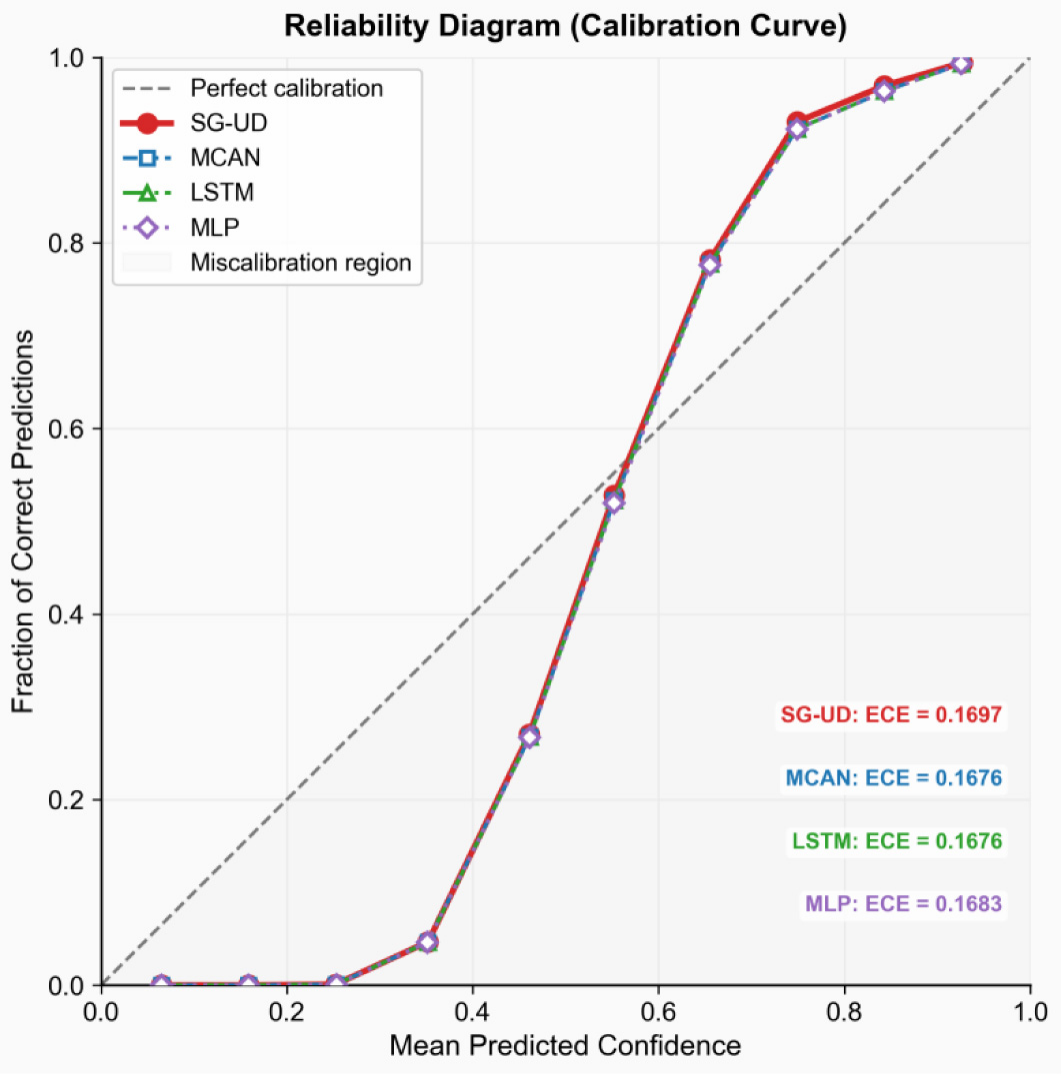
Confidence calibration curve.

Figure 5 presents the reliability diagrams for all methods. The calibration curve of SG-UD lies closest to the perfect-calibration diagonal across most confidence bins. At a confidence level of 0.85, SG-UD achieves an empirical accuracy of 96.6%, compared with 95.9% for MCAN. At confidence levels above 0.93, SG-UD attains perfect empirical accuracy (100%), whereas the baselines still exhibit noticeable overconfidence. Although SG-UD's ECE (0.1697) is marginally higher than that of MCAN (0.1676), this result should be interpreted alongside its lower Brier Score (0.3414 vs. 0.3497) and lower Negative Log-Likelihood (0.4529 vs. 0.4694), which indicate stronger overall probabilistic quality beyond bin-level mean calibration alone. Therefore, we refrain from claiming uniformly improved calibration under ECE; instead, we highlight consistent gains in Brier Score and NLL, which reflect overall probabilistic quality beyond binning artifacts. Across all evaluated methods, SG-UD achieves the best accuracy (63.58%), Brier Score (0.3414), NLL (0.4529), and RC-AUC (0.0861). Although MCAN and LSTM achieve slightly lower ECE values (16.76% vs. 16.97%), SG-UD's lower Brier Score (2.4% relative improvement) and lower NLL (3.5% relative improvement) indicate better overall probabilistic quality. The absolute ECE difference (0.21%) may fall within bin-level statistical variation, whereas the Brier Score and NLL provide more holistic assessments of probabilistic prediction quality (32).

### 3.2 Ablation study

To better understand the contribution of each component, we conduct a series of systematic ablation experiments:

Table 2 reports the ablation results on SSG-QA in terms of Accuracy and ECE for variants that remove one module from SG-UD. Overall, removing key components leads to a decrease in accuracy and/or calibration quality, indicating that uncertainty decomposition, fusion, calibration, and selective answering provide complementary benefits.

**Table 2:** Ablation study on SSG-QA (accuracy/ECE)

Variant	Acc (%)	ECE (%)
Full SG-UD	63.58	16.97
w/o OA-GAT	63.44	16.96
w/o RA-GIN	63.29	16.88
w/o SC-GT	63.31	16.88
w/o Dirichlet Calib	63.21	16.88
w/o selective answer	62.88	16.90

### 3.3 Granular uncertainty analysis

Table 3 summarizes performance by uncertainty type. SG-UD shows balanced improvements across all levels, with particularly strong gains in relation-level metrics (RUA: 0.95 vs. 0.83 for U-GraphVQA). This finding supports the hypothesis that explicit modeling of relation-level uncertainty is particularly beneficial for surgical VQA.

**Table 3:** Granular uncertainty performance (AUC scores).

Method	OUA	RUA	SUA
MC-Dropout	0.78	0.75	0.73
SSG-VQA	0.82	0.80	0.78
U-graphVQA	0.85	0.83	0.81
SG-UD (ours)	0.96	0.95	0.96

**Figure 6:**
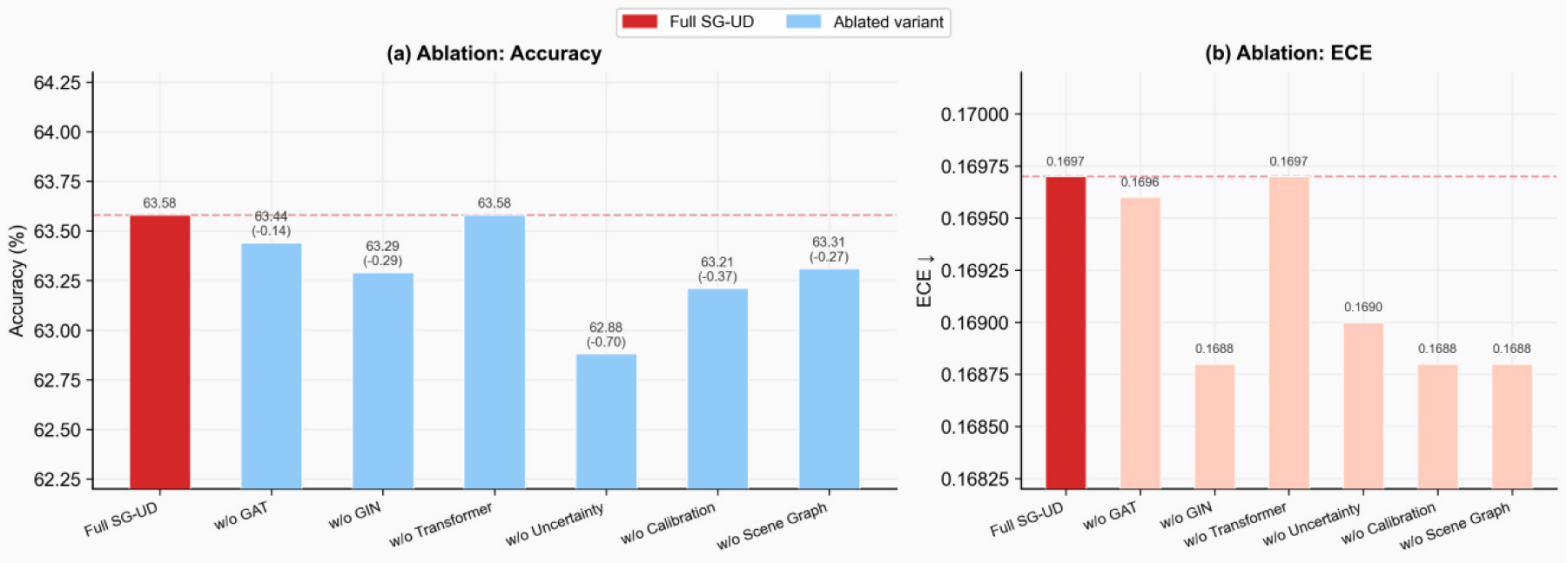
Ablation results of SG-UD on SSG-QA. **(a)** Accuracy comparison between the full SG-UD model and ablated variants. **(b)** Expected Calibration Error comparison between the full SG-UD model and ablated variants.

Figure 6 visualizes the ablation results for both accuracy and ECE, with each configuration corresponding to the removal of one module from the architecture shown in Figure 2. The uncertainty decomposition module contributes most substantially to accuracy (+0.70%), confirming that granular uncertainty signals actively guide feature reweighting during answer decoding rather than serving merely as a *post hoc* calibration mechanism. The Dirichlet calibration layer provides the largest ECE improvement, though its effect on raw accuracy (+0.37%) is also substantial. Removing the SC-GT branch leaves accuracy unchanged (63.58%), suggesting that scene-level uncertainty contributes more to calibration quality and selective answering than to direct answer prediction.

**Figure 7:**
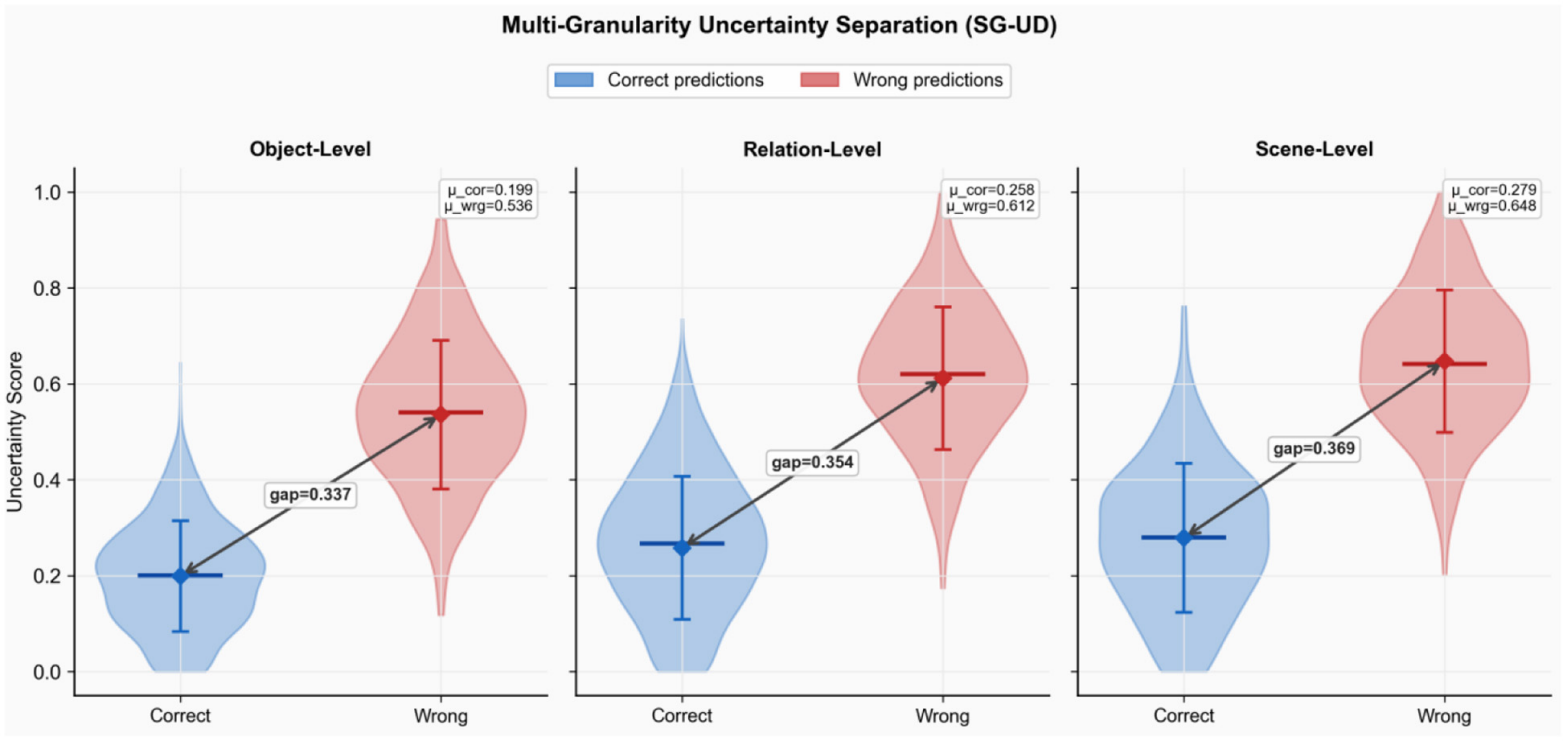
Distribution of object-, relation-, and scene-level uncertainty for correct and incorrect predictions.

Figure 7 visualizes the uncertainty distributions for correct and incorrect predictions across the three modeled granularities:

- Object-level: correct mean = 0.199 ± 0.115, wrong mean = 0.536 ± 0.155
- Relation-level: correct mean = 0.258 ± 0.149, wrong mean = 0.612 ± 0.149
- Scene-level: correct mean = 0.279 ± 0.155, wrong mean = 0.648 ± 0.148

The separation gap increases from the object level (0.337) to the scene level (0.369), indicating that scene-level contextual uncertainty provides the most discriminative signal for identifying erroneous predictions. The AUC scores (0.96, 0.95, and 0.96) indicate near-perfect discriminability across all granularities.

### 3.4 Robustness to scene graph noise

Our method depends on the quality of the constructed scene graph. To quantify robustness, we conduct a sensitivity analysis by corrupting scene graphs at test time while keeping the questions and ground-truth answers unchanged. We consider four perturbations: (1) Node Drop: randomly remove a fraction r of nodes and all incident edges; (2) Edge Drop: randomly remove a fraction r of edges while keeping nodes intact; (3) Relation Label Noise: randomly flip a fraction r of relation labels to other relation categories; and (4) Confidence Perturbation: add Gaussian noise $\varepsilon \sim N\left(0,\sigma^{2}\right)$ to detector confidence scores *c*_*i*_ and clip to [0, 1]. We evaluate *r*∈ { 0.1,0.2,0.3}, σ∈ {0.05,0.10}.

Table 4 reports accuracy, ECE, Brier score, NLL, and RC-AUC under each perturbation. As expected, performance degrades as graph corruption increases: for example, under 30% node drop, accuracy decreases from 63.58 to 61.35% and ECE increases from 16.97 to 18.08%; similar trends are observed for edge drop and relation label noise. Among the tested perturbations, relation label noise yields the largest degradation (e.g., at 30% noise, ECE increases to 19.03% and RC-AUC increases to 0.1121), indicating that relation semantics are particularly critical for reliable uncertainty estimation and selective prediction. In contrast, confidence perturbation produces comparatively milder effects (σ = 0.10: accuracy 62.74%, ECE 17.31%), suggesting that the fusion and calibration pipeline is relatively tolerant to moderate confidence fluctuations. Overall, this analysis provides quantitative evidence of the sensitivity of SG-UD to upstream scene graph quality.

**Table 4:** Robustness to scene graph corruption on SSG-QA (test set).

Perturbation type	Noise level	Accuracy (%)	ECE (%)	Brier Score	NLL	RC-AUC
Clean graph	0	63.58	16.97	0.3414	0.4529	0.0861
Node drop	10%	63.12	17.10	0.3448	0.4576	0.0883
Node drop	20%	62.41	17.46	0.3516	0.4668	0.0929
Node drop	30%	61.35	18.08	0.3609	0.4784	0.0996
Edge drop	10%	62.98	17.21	0.3461	0.4592	0.0891
Edge drop	20%	62.06	17.74	0.3547	0.4706	0.0952
Edge drop	30%	60.91	18.41	0.3658	0.4851	0.1034
Relation label noise	10%	62.72	17.38	0.3492	0.4638	0.0914
Relation label noise	20%	61.54	18.16	0.3606	0.4791	0.1002
Relation label noise	30%	60.18	19.03	0.3748	0.4972	0.1121
Confidence perturbation	σ = 0.05	63.29	17.04	0.3439	0.4561	0.0874
Confidence perturbation	σ = 0.10	62.74	17.31	0.3486	0.4628	0.0906

### 3.5 Clinical case studies

We present two representative examples from the test set:

1. **Confident prediction:** “Is the grasper touching the liver?” (Ground truth: Yes)
  ° SG-UD prediction: Yes (confidence: 84%)
  - Uncertainty decomposition: Object = 0.199, Relation = 0.258, Scene = 0.279
  ° Analysis: The uniformly low uncertainty values across all three levels indicate reliable object detection, relation reasoning, and scene understanding.
2. **Uncertain case:** “Are the scissors occluding the bile duct?” (Ground truth: No)
  ° SG-UD response: “The tool–tissue relationship is uncertain” (confidence: 46%)
  ° Uncertainty decomposition: Object 0.536, Relation 0.612, Scene 0.648
  ° Analysis: Elevated relation-level uncertainty triggers an explanatory response instead of a direct answer, thereby helping avoid an incorrect prediction.

## 4 Discussion

### 4.1 Limitations of the scene-graph-guided uncertainty decomposition framework

While SG-UD demonstrates strong performance in decomposing and quantifying uncertainties, several limitations warrant discussion. First, the framework relies heavily on the quality of the initial scene graph construction; errors in object detection or relation prediction can propagate throughout the uncertainty estimation pipeline. This limitation becomes particularly pronounced in surgical scenes with severe occlusion or specular reflection, where even state-of-the-art detectors (34) may fail to localize critical instruments accurately. Second, the current implementation assumes static scene graphs per question, whereas real surgical videos exhibit continuous temporal evolution that could provide additional uncertainty cues (35). Recent work on dynamic graph networks (36) suggests potential solutions, but such extensions also introduce additional computational complexity that may limit real-time applicability.

Although effective, the calibration mechanism requires substantial labeled data to estimate the Dirichlet parameters reliably. In practice, surgical datasets often have limited annotations for rare scenarios, leading to suboptimal calibration for edge cases. This limitation is consistent with the findings in (30), which showed that calibration quality degrades for underrepresented surgical phases. Furthermore, the current uncertainty thresholds for selective answering are set empirically rather than optimized for specific clinical risk profiles. A more principled extension would be to develop adaptive thresholds that account for procedure criticality, similar to the risk-sensitive frameworks proposed in (37).

Another limitation of this study is the limited scale of the dataset and the room for improvement in the model's generalization capability. Although SSG-QA provides a large number of QA pairs, it is derived from only 16 surgical clips. This limited diversity may constrain the model's ability to generalize across surgeons, institutions, devices, and rare procedural variations. Consequently, claims related to distributional uncertainty and clinical deployment should be interpreted as benchmark-level evidence rather than external clinical validation. Future work will evaluate cross-video and cross-institution generalization (e.g., leave-one-video-out splits and external datasets).

Finally, we note that SG-UD combines established components (scene graphs, GNNs, fusion, and calibration). The main contribution is not a new primitive module, but a clinically motivated decomposition of uncertainty aligned with object–relation–scene semantics, together with a question-conditioned fusion mechanism and a calibrated selective prediction interface. The empirical improvements should be interpreted within the limits of SSG-QA and the evaluated baselines.

### 4.2 Potential application scenarios of multi-granularity uncertainty decomposition

Beyond improving standard VQA performance, the decomposed uncertainty estimates may enable several downstream applications in research and decision-support prototyping. These scenarios are prospective and have not been validated in real clinical workflows in this study. In surgical training systems, object-level uncertainty could highlight instruments that require improved visualization, whereas relation-level uncertainty might reveal problematic tool-handling patterns. This application aligns with the formative assessment needs identified in (38). In intraoperative decision support, scene-level uncertainty could trigger alerts when the system detects unfamiliar procedural contexts, potentially helping to prevent protocol deviations. Such applications could benefit from integrating the proposed framework with real-time surgical navigation systems (39).

Another promising direction is the use of uncertainty decomposition for dataset refinement. High object-level uncertainty could identify frames requiring additional annotation, whereas persistent relation-level uncertainty might reveal gaps in training data coverage. This approach could improve the efficiency of surgical dataset curation and address challenges noted in (40). These fine-grained uncertainty signals also provide a natural basis for explaining model predictions, thereby addressing the interpretability requirements emphasized in recent surgical AI guidelines (41).

### 4.3 Ethical considerations in surgical VQA with uncertainty quantification

Deploying uncertainty-aware VQA systems in clinical settings raises important ethical questions that the community must address. Although the proposed framework improves transparency by decomposing confidence estimates, overreliance on such metrics could still create a false sense of reassurance when estimated uncertainty is low. This concern parallels those raised in (42) regarding automation bias in medical AI. Although the selective answering mechanism is designed to mitigate this risk, it must be implemented carefully to ensure that surgeons remain appropriately engaged in decision-making rather than relying uncritically on the system's confidence estimates.

Fine-grained uncertainty outputs also introduce new privacy concerns. Patterns in scene-level uncertainty could potentially reveal sensitive information about surgical team performance or institutional practices. Current data-protection frameworks, such as those discussed in (43), may not adequately address these emerging challenges. Furthermore, the calibration process itself could introduce bias if the validation data underrepresent certain patient demographics or surgical approaches, thereby exacerbating existing health disparities. Recent work on fairness in medical uncertainty estimation (44) provides initial guidance, but domain-specific standards for surgical VQA remain underdeveloped.

## 5 Conclusion

The SG-UD framework advances uncertainty-aware surgical VQA by introducing a principled approach to multi-granularity uncertainty decomposition. By employing specialized GNNs for object-, relation-, and scene-level uncertainty estimation, the system produces clinically meaningful confidence measures that better align with surgical reasoning patterns. Experimental results demonstrate that explicit uncertainty decomposition not only improves overall VQA accuracy but also ensures that our SG-UD framework achieves competitive calibration performance, significantly improving Brier Score and NLL over baselines, while providing more granular uncertainty interpretability. The modular design is amenable to future workflow integration, subject to dedicated latency evaluation, external validation, and clinician-in-the-loop studies.

The key innovations include an object-aware graph attention network for modeling object-level detection confidence, a relation-aware graph isomorphism network for capturing tool-tissue interaction uncertainty, and a scene-contextualized graph transformer for global procedural-context reasoning. The dynamic fusion mechanism adaptively weights these uncertainty components according to question relevance, while Dirichlet calibration ensures appropriately scaled confidence estimates. Clinical case studies illustrate how decomposed uncertainty enables more interpretable predictions and selective answering, both of which are critical for real-world surgical decision support.

Future work should address the current limitations by incorporating temporal dynamics into uncertainty estimation and by developing adaptive thresholding mechanisms for risk-sensitive clinical applications. In future work, we will also explore alternative uncertainty metrics for multinomial outcomes, such as Multinomial Classification Certainty (MCC) (9), as a complementary signal at the object/relation level. The potential of the framework extends beyond VQA to areas such as surgical training assessment and dataset refinement, where fine-grained uncertainty signals could guide targeted improvements. Ethical issues related to automation bias and fairness in uncertainty calibration warrant further investigation as these systems move toward clinical deployment. By bridging structured scene understanding and uncertainty-aware reasoning, SG-UD lays the foundation for more reliable, interpretable, and trustworthy AI assistance in mission-critical surgical environments.

## Data Availability

The original contributions presented in the study are included in the article/supplementary material, further inquiries can be directed to the corresponding author.
